# Gender Differences in the Association Between Leisure Activity in Adulthood and Cognitive Function in Old Age: A Prospective Longitudinal Population-Based Study

**DOI:** 10.1093/geronb/gbx170

**Published:** 2017-09-04

**Authors:** Linda B Hassing

**Affiliations:** Department of Psychology, and Centre for Ageing and Health—AgeCap, University of Gothenburg, Gothenburg, Sweden

**Keywords:** Cognitive aging, Cognitive reserve, Lifecourse approach

## Abstract

**Objectives:**

To examine the long-term association between leisure activities in adulthood and cognitive function in old age while recognizing gender differences in activity profiles.

**Methods:**

The sample included 340 cognitively healthy twins enrolled in the OCTO-Twin Study, a longitudinal study on cognitive aging. Leisure activity was measured in midlife and cognitive function in old age (mean age 83). Leisure activities covered the domains of domestic, intellectual–cultural, and self-improvement activities. The cognitive assessments comprised 5 measurement occasions (2-year intervals) covering verbal ability, spatial ability, memory, and speed. The association between leisure activity and cognitive function was estimated separately for the genders using growth curve models, adjusting for age and education.

**Results:**

Men and women had the same level of total leisure activity but differed in activity profiles and in the associations between activity and cognitive function. Higher engagement in self-improvement among men was related to higher level of cognitive functioning. Among women, intellectual–cultural activity was related to better verbal ability and memory. Concerning trajectories of cognitive function, domestic activity among men was related to less decline in speed, whereas for women it was related to steeper decline in spatial ability and memory. Further, higher intellectual–cultural activity among women was related to steeper decline in memory.

**Discussion:**

Cognitively stimulating activities (i.e., self-improvement and intellectual–cultural), might increase cognitive reserve whereas less cognitively stimulating activities (i.e., domestic) do not. Gender differences should be considered when examining lifestyle factors in relation to cognitive aging.

Leisure activities are activities free from duties of everyday life. It has been suggested that some types of leisure activities may play a role protecting cognitive health in aging, an issue of great concern for many people. One theoretical account for this association is the cognitive reserve hypothesis which states that cognitively stimulating activities during longer periods contribute to cognitive reserve that may delay onset of cognitive diseases such as dementia (Y. Stern, 2012). The main objective of this study was to examine the long-term association between different types of leisure activities in adulthood and cognitive function in old age. Further, as men and women may have different activity profiles, the study explored these differences in relation to cognitive aging.

Much research in the past decade has been dedicated to examine associations between different types of leisure activities and cognitive aging (for reviews see Bielak, 2010; Hertzog, Kramer, Wilson, & Lindenberger, 2008). There is a growing consensus that leisure activities in form of physical activity and cognitively stimulating activities contribute to prevention of dementia according to several systematic reviews (Fallahpour, Borell, Luborsky, & Nygård, 2016; C. Stern & Munn, 2010; Wang, Xu, & Pei, 2012). While these reviews have come to much the same conclusion, methodological problems have been identified in many of the studies included in the reviews which may call the validity of the conclusion into question. One methodological weakness of great significance relates to the short follow-up time between assessment of leisure activities and the outcome assessments of dementia or cognitive function. Given the prolonged prodromal phase of dementia, which may be as long as 20 years (Elias et al., 2000), with subtle cognitive and behavioral changes, a long follow-up time is crucial to reduce the risk of confounding. Thus, findings based on studies with short follow-up time might reflect reverse causation, as it is likely that leisure activities, especially those cognitively demanding, might become less frequent as an effect of prodromal dementia.

The problem of reverse causation has been recognized for some time in this research field (Gow, Corley, Starr, & Deary, 2012; Gow, Mortensen, & Avlund, 2012; Hertzog et al., 2008). In fact, reversed causality was demonstrated by Sörman, Sundström, Rönnlund, Adolfsson, and Nilsson (2014) who showed that the association between leisure activities and risk of dementia was dependent on the length of the follow-up time, such that only the shortest follow-up time (1–5 years) resulted in significant association, whereas no association was found with the longest follow-up time (6–15 years). This shows that the length of the follow-up time is of great significance in studies that aim at finding precursors to a disease, such as dementia, with a prolonged preclinical phase. In the present study, a lifecourse approach was used to examine the long-term association between leisure activities and cognitive aging by relating leisure activities before age 40 (reported in midlife) to cognitive performance and decline in old age. It is believed that by separating these measures by more than two decades there is a better chance to avoid reverse causation. Recent notions (Anderson & Craik, 2017; Gow, Pattie, & Deary, 2017) highlight the need to have a lifecourse approach when examining determinants of cognitive aging, partly because in this way, critical periods in life can be revealed that may be especially influential, and partly because by studying predictors in a long-term perspective, cause–effect relationships can be clarified.

Studies that have had a lifecourse approach, have found evidence that various leisure activities in adulthood or midlife are associated with better cognitive health in old age. For example, cognitively and socially engaging activities in midlife among men (Carlson et al., 2008), and intellectual/cultural activities in adulthood among women (Crowe, Andel, Pedersen, Johansson, & Gatz, 2003), were found to reduce risk of dementia 20–40 years later, in twins discordant for onset. Further, midlife mental and political activities were found to be significantly related to better cognitive function 20 years later (Kåreholt, Lennartsson, Gatz, & Parker, 2011). These findings give support to the cognitive reserve hypothesis by showing a long-term association between cognitively stimulating activities in adulthood and midlife and cognitive function in old age. Further, they also indicate that these associations may vary between men and women. This aspect has not gained much attention in the research literature (Bielak, 2010). Patterns of leisure activities may differ between men and women given differences in roles and responsibilities in many cultures, relating to, for example, life-span occupational opportunities (women taking care of family and home to a greater extent than men, whereas men work outside the home), therefore gender differences will be explored in the present study.

Taken together, there are only a few studies examining the impact of leisure activity on cognitive aging that meet the strict criteria so that reverse causation can be excluded. These studies give some support to the cognitive reserve hypothesis, in terms of lower dementia risk. Dementia is commonly used as an outcome variable, whereas relatively few studies have used more fine grained analysis of cognitive function, where cognitive function is examined in terms of different abilities or domains, for example, crystallized and fluid, and varies along a continuous scale. The need to understand the impact of leisure activity in relation to the different cognitive domains was noted by Bielak (2010), as there is some evidence that cognitive abilities may be differentially affected (Bielak, Gerstorf, Anstey, & Luszcz, 2014). Also, there is some evidence that only levels of cognitive function are affected and not the trajectories (Bielak, Anstey, Christensen, & Windsor, 2012).

The present study has several important methodological strengths related to its design that may help clarify the role of leisure activities in cognitive reserve and cognitive aging. Thus, (a) relating to reverse causation, the present study analyzed information on leisure activity before age 40 in relation to cognitive outcomes in old age; (b) regarding the different cognitive domains, the cognitive measures included seven tests reflecting domains of verbal ability, spatial ability, long-term memory, and speed; (c) regarding trajectories of cognitive change, cognitive function was examined five times across 8 years, at 2-year intervals, and was analyzed by separating between-person differences and within-person change, using mixed growth curve models; and (d) relating to differences between men and women in activity profiles, all models were runned separately for men and women.

## Methods

### Study Design and Data Sources

This study is based on two datasets data from the Swedish Twin Registry that have been linked; a dataset on a survey from 1967, and a dataset from a longitudinal study on cognitive health (the Origins of Variance in the Old-Old; OCTO-Twin). The Swedish Twin Registry was established in the late 1950s, initially to study smoking and alcohol consumption in relation to risk of cancer and cardiovascular diseases (Lichtenstein et al., 2002, 2006). In 1967, a survey was administered including questions about leisure activities, as well as many other aspects such as health and diseases, smoking and alcohol habits, weight, and height. In the early 1990s the longitudinal study OCTO-Twin was started, with a special focus on cognitive health (McClearn et al., 1997). Most of the participants in the OCTO-Twin study had participated in the survey in 1967. For the purpose of the present study, information on leisure activity from the survey in 1967 and information on the cognitive tests from the longitudinal OCTO-Twin study has been linked.

### Participants

The OCTO-Twin study is based on a sample of 702 individuals, in 351 same-sex pairs, aged 80 years and older, that was drawn from the oldest cohort (born between the years 1893 and 1911) of the Swedish Twin Registry. The representativeness of the sample was supported by a study which compared one randomly chosen member of each dyad to a population-based sample of Swedish singletons of the same age (Simmons et al., 1997). Given the purpose of the present study, that is to examine the association between leisure activity reported in midlife and cognitive function in old age, in a cognitively healthy sample, the following individuals were excluded: (a) those who did not have valid information concerning leisure activity in the midlife survey in 1967 (*n* = 107), (b) those who did not have valid cognitive data (*n* = 68), and (c) those who received a dementia diagnosis during the longitudinal follow-up (*n* = 187) according to the Diagnostic and Statistical Manual of Mental Disorders (3rd ed., rev; American Psychiatric Association, 1987). The total sample at the first wave of the longitudinal study includes 340 individuals (115 men and 225 women). The background characteristics of the study sample are presented in Table 1. There were no differences between men and women concerning age or general cognitive status, as indicated by the Mini-Mental State Examination (Folstein, Folstein, & McHugh, 1975). There was a difference in educational attainment; men had somewhat higher education [*t*(338) = 2.08, *p* = .04].

**Table 1.** Sample Characteristics

	All, *M* (*SD*)	Men, *M* (*SD*)	Women, *M* (*SD*)
*N*	340	115	225
Age in 1967	57.6 (2.8)	57.3 (2.8)	57.8 (2.9)
Range	54–67	54–67	54–67
Age in 1991	83.0 (2.7)	82.8 (2.6)	83.1 (2.7)
Range	80–93	80–92	80–93
Education (years in school)	7.4 (2.5)	7.8 (3.1)	7.2 (2.1)^*^
Range	0–20	3–20	0–14
Mini Mental State Examination in 1991	27.9 (2.0)	27.8 (2.2)	28.0 (2.3)
Range	19–30	19–30	19–30

* *p* < .05.

Concerning those who were excluded from the study, attrition analyses showed that this group was older [84 vs 83 years: *t*(700) = 4.78, *p* < .01] and had lower educational level [6.9 vs 7.4 years: *t*(700) = 3.50, *p* < .01], whereas there was no difference concerning sex (*p* = .79). Those who were excluded because of no cognitive data or because of dementia did not differ from those who participated in regard to total leisure activity (*p* = .28), or the domains of intellectual–cultural and self-improvement (*p* = .34), whereas they had slightly lower levels in the domain of domestic activity [1.0 vs 1.1: *t*(614) = 2.26, *p* = .02].

All participants were informed about the study in accordance with the ethics committee of the Karolinska Institutet, Sweden, the Swedish Data Inspection Board, and the institutional board at the Pennsylvania State University, USA.

### Leisure Activity

The measure of leisure activity is based on a questionnaire that was sent out to individuals registered in the Swedish Twin Registry in 1967. The measure included questions regarding participation in leisure activities phrased as “If you look back in time, which were your main leisure activities before the age 40.” The items were the following 11 activities (yes or no); house and garden, home and family, reading, radio and TV, social visits, cultural activities (such as theatre and cinema), clubs and organizations, studies, playing sports, outdoor activities, and hobbies. Three activity domains were composed, based on a principal component analysis, in accordance with the procedure reported by Crowe et al. (2003). These domains were domestic activities (two items: house and garden, home and family; range 0–2), intellectual–cultural activities (four items: reading, radio and TV, social visits, cultural activities; range 0–4), and self-improvement (four items: clubs and organizations, studies, playing sports, outdoor activities; range 0–4). One item (hobbies) was discarded as it did not load highly on any one of the factors.

### Cognitive Tests

The participants were investigated in their home, by experienced registered nurses, specifically trained for the study, and continuously supervised. The longitudinal design encompassed at maximum five measurement occasions at 2-year intervals beginning in 1991–1993. The cognitive test battery was designed to represent the domains of fluid and crystallized abilities and specific abilities such as memory, spatial ability, and speed. Principal component analyses were used to construct latent factors from the individual tests within each cognitive ability reflecting verbal ability, spatial ability, long-term memory, and speed. For the ease of presentation cognitive scores were transformed into a *t*-score metric with mean of 50 and standard deviation of 10. Number of participants completing each test across the five waves is presented in Table 2.

**Table 2.** Participation (*n*) and Valid Cognitive Data, *n* (%) Across Gender, Tests, and Waves (*N* = 340)

	Wave 1	Wave 2	Wave 3	Wave 4	Wave 5
Men	115	98	77	55	41
Retained from the original sample/prior wave, %	—	85/85	67/79	48/71	36/75
Verbal ability	101 (88)	82 (84)	66 (86)	38 (69)	26 (71)
Spatial ability	100 (87)	80 (82)	58 (75)	38 (69)	24 (59)
Memory	91 (79)	63 (64)	49 (64)	26 (47)	13 (32)
Speed	69 (60)	63 (64)	47 (61)	29 (71)	—^a^
Women	225	186	158	131	112
Retained from the original sample/prior wave, %	—	83/83	70/85	58/83	50/85
Verbal ability	199 (88)	150 (81)	125 (79)	96 (73)	76 (68)
Spatial ability	192 (85)	146 (78)	129 (82)	104 (79)	78 (70)
Memory	180 (80)	136 (73)	108 (68)	79 (60)	57 (51)
Speed	145 (64)	112 (60)	94 (59)	69 (53)	—^a^

a The Speed measure was not used in Wave 5.

#### Verbal ability

To measure verbal ability two tasks from the Wechsler Adult Intelligence Scale (Wechsler, 1991) were used; the Swedish version of the Information Task, including questions of general knowledge (Jonsson & Molander, 1964), and the verbal meaning test (Dureman & Sälde, 1959), which required finding a synonym to match a target word.

#### Spatial ability

The Block Design test (Dureman & Sälde, 1959) was used. In the test, the participant is presented with red and white blocks and several patterns written on cards. The task is to reproduce the pattern shown on the cards by assembling the proper blocks and arranging them to form the design shown on the card.

#### Memory

Three tests reflecting long-term episodic memory functioning were used. The Prose Recall test (a Swedish version of the Logical Memory in the Wechsler Memory Scale), The Thurstone’s Picture Memory test (Thurstone & Thurstone, 1949), and Memory-in-Reality (Fiske & Gatz, 2007; Johansson, 1988). In the prose recall test, the task is to recall a short story. In the Thursone’s test, task is to recognize the target drawn picture among several pictures. The memory-in-reality test is a free recall of 10 real objects.

#### Speed

Two tests measured speed; the Symbol-Digit task, a modified version of the speeded Digit-Symbol Substitution test from the Wechsler Adult Intelligence Scale-Revised (Wechsler, 1991), and the Perceptual Speed test from a Swedish test battery (Dureman & Sälde, 1959). The Symbol-Digit task, requires a verbal (rather than written) response that a digit matched or did not match a symbol. In the perceptual speed test, the task is to detect an identical item, among five alternatives, to one item at the far left as fast as possible. This task was not used in Wave 5.

#### Mini-mental state examination

The MMSE is a measure of global cognitive functioning. The task reflects orientation, memory, attention, ability to follow verbal and written commands, writing, and copying. The maximum score is 30, in this study any score greater than or equal to 24 points indicated a normal cognition.

### Data Analyses

To test the hypothesis that leisure activities are related to cognitive function, linear mixed effects growth models were conducted separately for men and women where the three activity domains were related to the four cognitive domains. Cognitive decline was measured by slope from the linear mixed effects models with leisure activity domains as the independent variables and the longitudinally measured cognitive domain scores as the dependent variables. Mixed effects growth models were fitted to estimate individual-level change and predictors of change while properly accounting for the dependency associated with twin pair status.

The multilevel model is characterized by a fixed part which contains average effects for the intercept (initial status) and slope (rate of change over time) and a random part which contains individual differences (variance) in the intercept, slope, and the within person residual. The models were tested using the missing at random assumption for missing cognitive outcomes. A three-level linear growth model is composed of a Level 1 component of individual outcomes over time, a Level 2 component which models individual fixed and random effects of initial status and change over time (person-level covariates can be added at this level), and the Level 3 component which models variance associated with twin-pair status. This is done to handle the fact that a twin sample is used to draw conclusions about nontwins in which higher intraclass correlations are expected for monozygotic than for dizygotic twins. Thus, a three-level structure is characterized by longitudinal measurements nested within individuals who are nested within groups (twin dyad).

First, before any covariates were added to the models, unconditional growth models were fitted for the cognitive tests. Then, to test the long term associations between leisure activities in adulthood and cognitive function in old age, each activity domain (as continuous variable) was tested in relation to the four cognitive domains, adjusted for age and education, and run separately for men and women. The intercept was centered at age 83, and education at 7 years.

## Results

### Gender Differences in Leisure Activities

**Figure 1.**
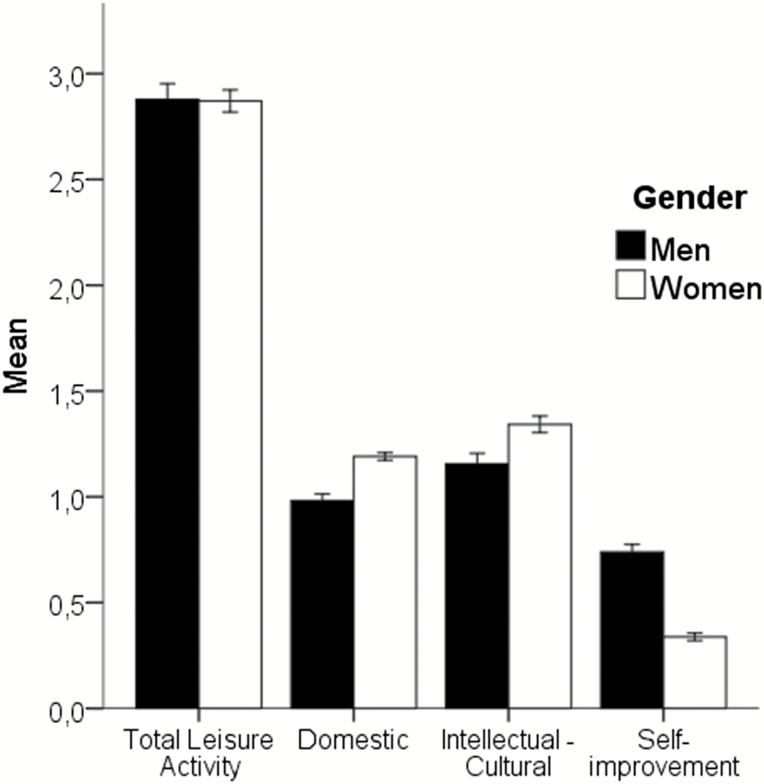
Total leisure activity and specific domains reported in midlife (means and standard errors).

Men and women had the same total activity level (*p* = .97) but they reported different activity profiles (Figure 1). Women were more active than men within the domain of domestic activities [*t*(338) = 2.78, *p* < .01], whereas men were more active than women in self-improvement [*t*(338) = 4.96, *p* < .01]. Women reported higher intellectual–cultural activities compared to men, although this difference was not statistically significant (*p* = .21).

### Leisure Activities in Relation to Cognitive Functioning

**Figure 2.**
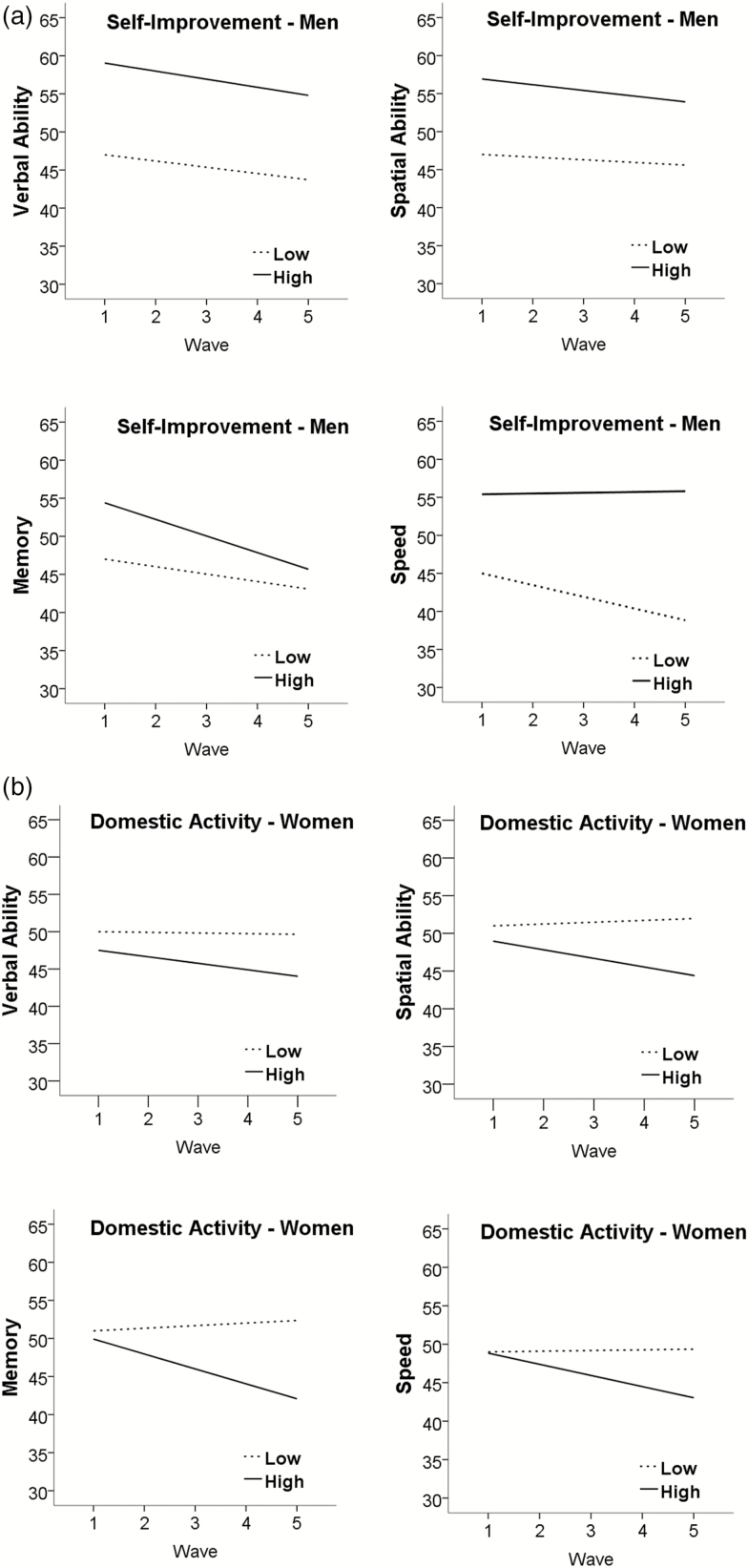
(a) Self-improvement and cognitive function—men. *Note.* Estimated means (*t*-scores) of linear mixed models adjusted for age and education. (b) Domestic activity and cognitive function—women. *Note.* Estimated means (*t*-scores) of linear mixed models adjusted for age and education.

For men, the results of the association between leisure activity and later cognitive functioning are presented in Table 3 and illustrated in Figure 2a (self-improvement only). Higher self-improvement was significantly related to higher performance in verbal ability (*β* = 3.01, *SE* = 0.93, *p* < .01), spatial ability (*β* = 2.48, *SE* = 1.07, *p* = .02), and speed (*β* = 2.60, *SE* = 1.01, *p* < .01), and nonsignificantly to memory (*β* = 1.85, *SE* = 0.96, *p* = .06). No other associations were found except for the positive slope indicating increase in speed related to higher domestic activity (*β* = 1.24, *SE* = 0.41, *p* < .01).

**Table 3.** Parameter Estimates (Est.) and Standard Errors (*SE*) From the Growth Curve Models by Cognitive Domains; Men (*n* = 155)

		Verbal ability			Spatial ability			Memory			Speed		
Leisure activity		Est.	*SE*	*p*	Est.	*SE*	*p*	Est.	*SE*	*p*	Est.	*SE*	*p*
Domestic	Intercept	50.04	1.44	<.01	47.60	1.69	<.01	48.35	1.53	<.01	48.05	1.64	<.01
	Slope	−0.81	0.32	.02	−0.39	0.44	.38	−0.95	0.73	.20	−2.44	0.49	<.01
	Domestic	−0.46	1.13	.69	1.36	1.28	.29	−0.39	1.19	.74	−0.83	1.25	.51
	Slope × Domestic	−0.05	0.27	.85	−0.02	0.36	.96	−0.26	0.61	.68	**1.24**	**0.41**	**<.01**
Intellectual– cultural	Intercept	49.97	1.19	<.01	49.71	1.38	<.01	49.03	1.22	<.01	48.32	1.33	<.01
	Slope	−1.13	0.29	<.01	−0.40	0.39	.30	−1.69	0.65	.01	−1.24	0.52	.02
	Intellectual– cultural	−0.34	0.74	.65	−0.55	0.81	.49	−0.96	0.72	.19	−0.97	0.81	.24
	Slope × Intellectual– cultural	0.22	0.18	.22	−0.01	0.24	.96	0.42	0.40	.29	0.03	0.33	.94
Self-improvement	Intercept	47.37	1.06	<.01	47.29	1.24	<.01	46.51	1.17	<.01	45.38	1.19	<.01
	Slope	−0.82	0.27	<.01	−0.35	0.37	.35	−0.98	0.60	.11	−1.54	0.47	<.01
	Self-improvement	**3.01**	**0.93**	<**.01**	**2.48**	**1.07**	**.02**	1.85	0.96	.06	**2.60**	**1.01**	**.01**
	Slope × Self-improvement	−0.06	0.23	.80	−0.10	0.34	.78	−0.30	0.49	.55	0.41	0.36	.26

*Note*: The significance of the estimate 1.24 is indicated in the column for the p, that is, *p* < .01.

For women, the results are presented in Table 4 and illustrated in Figure 2b (domestic activity only). Higher involvement in intellectual–cultural activities was related to significantly higher performance in verbal ability (*β* = 1.10, *SE* = 0.43, *p* = .01), and memory (*β* = 1.05, *SE* = 0.49, *p* = .04) as well as steeper decline in memory (*β* = −0.67, *SE* = 0.26, *p* = .01). High involvement in domestic activities was related to significantly steeper decline in spatial ability (*β* = −0.69, *SE* = 0.32, *p* = .03), and memory (*β* = −1.15, *SE* = 0.48, *p* = .02).

**Table 4.** Parameter Estimates (Est.) and Standard Errors (*SE*) From the Growth Curve Models by Cognitive Domains; Women (*n* = 225)

		Verbal ability			Spatial ability			Memory			Speed		
Leisure activity		Est.	*SE*	*p*	Est.	*SE*	*p*	Est.	*SE*	*p*	Est.	*SE*	*p*
Domestic	Intercept	50.10	1.25	<.01	50.59	1.46	<.01	51.76	1.41	<.01	49.47	1.57	<.01
	Slope	−0.09	0.31	.78	0.24	0.41	.56	0.34	0.64	.60	0.09	0.53	.86
	Domestic	−1.25	0.93	.18	−1.02	1.11	.36	−0.54	1.04	.60	−0.08	1.17	.95
	Slope × Domestic	−0.39	0.24	.11	−0**.69**	**0.32**	**.03**	−**1.15**	**0.49**	**.02**	−0.77	0.41	.07
Intellectual– cultural	Intercept	47.12	0.81	<.01	48.71	0.96	<.01	49.13	0.91	<.01	48.91	1.03	<.01
	Slope	−0.49	0.24	.04	−0.13	0.31	.66	−0.06	0.47	.90	−0.46	0.42	.27
	Intellectual– cultural	**1.10**	**0.43**	**.01**	0.46	0.51	.37	**1.05**	**0.50**	**.04**	0.33	0.56	.55
	Slope × Intellectual– cultural	−0.02	0.13	.86	−0.29	0.17	.08	−0**.67**	**0.26**	**.01**	−0.21	0.23	.35
Self-improvement	Intercept	48.43	0.65	<.01	49.38	0.77	<.01	50.38	0.74	<.01	49.79	0.83	<.01
	Slope	−0.40	0.18	.03	−.49	0.25	.05	−0.74	0.38	.05	−0.82	0.32	.01
	Self-improvement	0.48	0.96	.61	−0.08	1.11	.94	0.34	1.08	.76	−1.27	1.21	.30
	Slope × Self-improvement	−0.36	0.26	.17	−0.11	0.34	.74	−0.62	0.56	.27	0.18	0.45	.69

## Discussion

The general aim of this study was to explore the role of leisure activities earlier in life in relation to cognitive aging while recognizing differences between men and women in activity profiles. This was done by examining the impact of different domains of leisure activities before age 40 as potential protection for cognitive function and decline in old age. The general pattern of the findings indicated that not all leisure activities have a protective effect on cognitive aging, and that there are gender differences in these relationships that need to be considered. More specifically, for men, the only activity domain that was related to cognitive function was self-improvement which was related to significantly higher levels of performance in verbal ability, spatial ability, and speed. There was only one significant effect related to trajectories, reflecting less decline in speed related to higher domestic activity. For women, higher intellectual–cultural activity was related to higher level of performance in verbal ability and memory as well as steeper decline in memory. Further, higher domestic activity was significantly associated with steeper decline in spatial ability and memory.

Before discussing the impact of adult leisure activities in relation cognitive aging the differences between men and women in activity profiles need to be recognized. First, when comparing the activity domains in Figure 1 with each other it should be noted that the domestic domain ranges between 0 and 2, whereas the other two domains range between 0 and 4. It is clear that there were gender differences in all three activity domains, where women reported significantly higher activities in the domestic domain, and significantly lower activities in the self-improvement domain, as compared to men. The difference in the intellectual–cultural domain was not statistically significant. It should be kept in mind that these activity reports are from the middle of the last century and as such do not necessarily generalize to men and women today, although it is well established that women still take greater responsibility for the family and home (domestic) as compared to men.

Concerning the association between leisure activities and cognitive aging, the finding among men that activities related to self-improvement were positively associated to cognitive functioning is in line with other findings (Carlson et al., 2008; Fallahpour et al., 2016; Kåreholt et al., 2011; C. Stern & Munn, 2010; Wang et al., 2012), and supports the cognitive reserve hypothesis, as some of these activities are characterized by cognitively stimulating and demanding activities such as engaging in studies. Other items included in the self-improvement factor, like playing sports and engagement in clubs and organizations, are activities that can involve complex social interactions between people, which are generally thought of as being especially cognitively demanding and stimulating, giving further support to the cognitive reserve hypothesis. The finding that self-improving activities did not have the same positive impact for women as for men was not expected. There is not a plausible theoretical account for this null-result. It can only be speculated that the lack of associations for women may be related to lack of statistical power as this type of activity was rarely reported by the women in this study, making it difficult to demonstrate associations.

For women, on the other hand, the positive impact of intellectual–cultural activities on levels of performance in verbal ability and in memory were also in line with other findings (Carlson et al., 2008; Fallahpour et al., 2016; Kåreholt et al., 2011; C. Stern & Munn, 2010; Wang et al., 2012), and can be seen as a support to the cognitive reserve hypothesis. For men, intellectual–cultural activities were not of importance at all for any cognitive ability in this study. Verbal ability is a part of the crystallized abilities, which are known to show greater plasticity as compared to the fluid abilities. Thus, verbal abilities can increase across the life-span as a result of educational attainment and other enriching experience such as reading and theatre and cinema visits. The observation that intellectual–cultural activities were not of importance for spatial ability and speed may be explained by similar line of reasoning, that is, that fluid abilities are more difficult to manipulate in general.

Concerning associations between leisure activities and change in cognitive function, clear effects on the trajectories were found among women, reflecting steeper cognitive decline in relation to higher domestic activities. How these associations are to be interpreted is not entirely clear. Considering that most women in this study were housewives, which implies taking care of the home and family, it is suggested that performing these activities as leisure activities as well, may result in less variation in activities in general and less time to participate in self-improving activities, which in this study were found to have a clear protective effect on cognitive function for men. It could be hypothesized that this may explain the steeper decline related to domestic activities found for women. For men, on the other hand, higher domestic activity was related to less decline in speed. In the light of the discussion above, it can be speculated that men, in contrast to women, gain from being active in domestic activities as this is an activity that extends the variation of activities, whereas that was probably not the case for many women. Another significant trajectory was found for intellectual–cultural activities in women, reflecting steeper decline in relation to higher activity levels earlier in life. Thus, even though higher intellectual–cultural activities were related to higher level of memory performance it was also related to steeper decline. This finding contradicts the results reported by Bielak and colleagues (2012), who found that only levels of cognitive function were affected by activity engagement and not the trajectories. However, according to the cognitive reserve hypothesis steeper decline is predicted when decline starts, as has been shown in studies on cognitive reserve and Alzheimer’s disease (Y. Stern, 2012). Similar findings were presented in a recent study on secular trends in cognitive aging, in which it was found that although later birth cohorts performed at a higher level, they also showed steeper decline (Thorvaldsson, Karlsson, Skoog, Skoog, & Johansson, 2017).

In general, there were rather few significant effects in this study. It should be noted that all individuals with a dementia diagnosis were excluded to obtain a cognitively intact sample with valid cognitive measures. One consequence of this was that the variation in the cognitive tests was restricted and the power to detect significant associations was reduced. On the other hand, the estimates were more stable. Although the analyses had sufficient power to detect differences in levels, the power to detect interaction effects, such as those between the slope and activity, is typically much lower. When inspecting the estimates for the interaction terms between the slopes and activity levels it can be seen that they are generally low, which implies that there was little impact on cognitive change in this study. Given the high age of this sample, many individuals can be considered to be in the terminal phase of life, resulting in terminal decline in cognitive functioning, which may have overshadowed the benefit of cognitive stimulation earlier in life. Analyses, not including the covariates of leisure activities, showed clear and significant negative trajectories in all the cognitive domains (results not presented), lending support to this notion. Another explanation may be that the effect of cognitive stimulation is primarily reflected in gains in levels of cognitive functioning and not in rate of cognitive decline. This has been suggested by several studies (Bielak et al., 2012; Hertzog et al., 2008; Hultsch, Hertzog, Small, & Dixon, 1999).

There are some methodological issues that need to be discussed. Relating the findings of the present study to the issue of reverse causation (Gow, Corley, et al., 2012; Hertzog et al., 2008), the findings can be considered to be robust against the threat of reverse causality as the measures on leisure activity and cognitive function were separated by more than two decades. At the same time, it should be noted that there was only one assessment of leisure activity; therefore, we do not know how activity patterns might have changed across the life-span. Although changes in leisure activities are to be expected in relation to entering old age, as a result of the multiple changes in life in general, greater consistency in leisure activities across adulthood, as compared to in old age, should be expected given that most people are rather habitual in their behavior. The tool included ten items reflecting the main leisure activities that people were engaged in. It should be noted that this measure, which was obtained in midlife, was a retrospective report on activities people were engaged in more than 10 years earlier, that is, before age 40. Retrospective reports are always subject to some errors as people may not remember things correctly. Further, a factor analysis was conducted to categorize the items into meaningful domains which resulted in three categories. It could be argued that there are other ways to conduct the categorization that would have resulted in other domains; however, this was believed to be the most correct way to form the domains. Another shortcoming of the instrument is that it does now allow for a precise quantification of each activity.

A limitation related to the design of the study is that the cognitive assessments were only conducted in old age, lacking a baseline assessment in midlife. This has implications to the conclusions of the study because it is likely that those who have better mental abilities are to a higher degree engaged in cognitively stimulating activities like self-improvement (Hertzog et al., 2008). The best way to control for the lack of baseline cognitive assessment was to use educational attainment as a proxy to general cognitive ability, thus, education was used as a covariate in all models. Another factor of concern that should be considered, as in all studies on older age groups, is the survival effect. First, it is clear that there has been a selective attrition in this study from midlife to old age related to survival, and further attrition across the five waves in old age. To note, among men only 36% of the original sample at Wave 1 survived into Wave 5, whereas the corresponding survival among women was 50%. There was also more missing cognitive data at the last wave among men as compared to women, especially in the measures of spatial ability (41% vs 30%) and memory (78% vs 49%). This differential survival, which reflects longer survival among women, and differential missing cognitive data, might have affected the findings of the current study primarily through loss of statistical power in the male sample. Second, by excluding all individuals with dementia, it needs to be kept in mind that the findings from this study can only be generalized to populations of cognitively healthy individuals.

The main strengths of the study are related to design- and methodological factors. The study sample is a population-based sample that gives strength to the generalizability of the results. The cognitive assessments included a range of validated tests that capture specific domains of cognitive functioning. The longitudinal design, including cognitive assessments every other year, across five time-points, makes it possible to examine both level of cognitive functioning and cognitive trajectories across time. The longitudinal assessments also allow for robust analyses of data as variance related to both within-individual change and between individual differences is analyzed. Finally, by using the information on leisure activity reported in midlife, as opposed to in old age, it is believed that the risk of reverse causation is minimized.

To summarize, there were not many associations between leisure activities and cognitive function, with the exception of positive effect of self-improvement for men, and intellectual–cultural activities for women, and negative effect of domestic activities for women; associations which were relatively stable across the cognitive abilities. One conclusion that can be drawn from this study, however, is that it is of importance to look at these associations separately for men and women, especially if there are substantial differences between the genders in the reported activities. Further, although the instrument was not optimal for mapping leisure activities, it managed to show some associations with cognitive function more than two decades later that may support the notion that engaging in cognitively stimulating activities can in the long run protect cognitive functions.

## Funding

This work was supported by the Bank of Sweden Tercentenary Foundation (P12-0567). The OCTO-Twin Study was supported by a grant from the National Institute of Aging (NIA: AG 08861) of the National Institutes of Health, and The Swedish Research Council for Health, Working Life and Welfare—Forte (Epilife FAS Center, and AgeCap 2013–2300).
